# Xanthine Oxidase Mediates Axonal and Myelin Loss in a Murine Model of Multiple Sclerosis

**DOI:** 10.1371/journal.pone.0071329

**Published:** 2013-08-08

**Authors:** Josephe Archie Honorat, Makoto Kinoshita, Tatsusada Okuno, Kazushiro Takata, Toru Koda, Satoru Tada, Takashi Shirakura, Harutoshi Fujimura, Hideki Mochizuki, Saburo Sakoda, Yuji Nakatsuji

**Affiliations:** 1 Department of Neurology, Osaka University Graduate School of Medicine, Suita, Osaka, Japan; 2 Department of Immune Regulation, Osaka University Graduate School of Medicine, Suita, Osaka, Japan; 3 Department of Neurology, National Hospital Organization Toneyama National Hospital, Toyonaka, Osaka, Japan; 4 Pharmaceutical Development Research Laboratories, Teijin Pharma Ltd., Tokyo, Japan; Research Inst. of Environmental Med., Nagoya Univ., Japan

## Abstract

**Objectives:**

Oxidative stress plays an important role in the pathogenesis of multiple sclerosis (MS). Though reactive oxygen species (ROS) are produced by various mechanisms, xanthine oxidase (XO) is a major enzyme generating ROS in the context of inflammation. The objectives of this study were to investigate the involvement of XO in the pathogenesis of MS and to develop a potent new therapy for MS based on the inhibition of ROS.

**Methods:**

XO were assessed in a model of MS: experimental autoimmune encephalomyelitis (EAE). The contribution of XO-generated ROS to the pathogenesis of EAE was assessed by treating EAE mice with a novel XO inhibitor, febuxostat. The efficacy of febuxostat was also examined in *in vitro* studies.

**Results:**

We showed for the first time that the expression and the activity of XO were increased dramatically within the central nervous system of EAE mice as compared to naïve mice. Furthermore, prophylactic administration of febuxostat, a XO inhibitor, markedly reduced the clinical signs of EAE. Both *in vivo* and *in vitro* studies showed infiltrating macrophages and microglia as the major sources of excess XO production, and febuxostat significantly suppressed ROS generation from these cells. Inflammatory cellular infiltration and glial activation in the spinal cord of EAE mice were inhibited by the treatment with febuxostat. Importantly, therapeutic efficacy was observed not only in mice with relapsing-remitting EAE but also in mice with secondary progressive EAE by preventing axonal loss and demyelination.

**Conclusion:**

These results highlight the implication of XO in EAE pathogenesis and suggest XO as a target for MS treatment and febuxostat as a promising therapeutic option for MS neuropathology.

## Introduction

Multiple sclerosis (MS) is an inflammatory demyelinating disease of the central nervous system (CNS) and is a leading cause of disability in young adults [1]. It is characterized by CNS autoimmunity that evokes a cascade of pathological processes, including demyelination and axonal injury, leading to impaired mobility in patients with MS [2], [3]. Reactive oxygen species (ROS) have been suggested to play an important role in the myelin and axonal impairments in MS[4]–[6]. Indeed, various oxidative stress markers are expressed in brain tissues from MS patients, particularly in apoptotic oligodendrocytes, axonal spheroids and degenerating neurons [7]–[9]. The production of ROS by macrophages/microglia is also increased in the CNS of experimental model of MS, namely, autoimmune encephalomyelitis (EAE) [4], [10]. In addition, several studies have reported that ROS scavengers, such as edaravone [11] and alpha-lipoic acid [12], produce therapeutic benefits in EAE, thereby supporting the notion that ROS play a role in the pathogenesis of MS.

In the context of inflammation, several distinct pathways can generate ROS and reactive nitrogen species (RNS); these include uncoupling of mitochondrial oxidative phosphorylation, induction of nitric oxide synthase (NOS), and the activation of nicotinamide adenine dinucleotide phosphate (NADPH) oxidase and xanthine oxidoreductase (XOR) systems [13], [14]. Among these catalyzing enzymes, the relationship between NADPH oxidase and ROS generation has been well characterized [15], with studies showing that NADPH oxidase is expressed in activated microglia and infiltrating macrophages in the active lesions of MS [16], [17]. By contrast, relatively little is known concerning the relationship between another ROS generating enzyme, XOR, and CNS autoimmunity. Xanthine dehydrogenase (XDH) and xanthine oxidase (XO) are inter-convertible forms of XOR, and the best characterized function of both enzymes is the conversion of hypoxanthine to xanthine and the conversion of xanthine to uric acid (UA) [18]. In addition to its involvement in the purine metabolism, there is growing evidence that XOR plays more diverse pathophysiological roles through the generation of ROS. Indeed, XOR produces various types of ROS, including superoxide, hydrogen peroxide and nitric oxide, by distinct mechanisms, and these ROS are involved in several types of tissue injury, such as acute lung injury, ischemia and reperfusion injury, and atherosclerosis [19]–[22]. Febuxostat is a thiazolecarboxylic acid derivative that is a novel, selective, non-purine inhibitor of XOR. It has potent and well-tolerated effects on uric acid synthesis, and its use for the treatment of gout was recently approved in several countries, including the US and throughout the European Union [23], [24]. Thus, the objectives of this study were to investigate the involvement of XOR in the pathogenesis of MS and to develop a potent new therapy for MS based on the inhibition of ROS.

## Materials and Methods

### Induction and Clinical Evaluation of EAE

EAE was induced in 8-week-old female SJL/J mice (Oriental Yeast Corp., Tokyo, Japan) by subcutaneous injection of 150 µg of PLP_139–151_ (Biologica, Nagoya, Japan) peptide emulsified in complete Freund’s adjuvant (CFA; Sigma, St. Louis, MO, USA ) supplemented with 400 µg of *Mycobacterium tuberculosis* (H37Ra strain; Difco Laboratories, Detroit, MI, USA) [11]. In non-obese diabetic (NOD)/ShiJcl mice, EAE was induced by immunization with MOG_35–55_ peptide (Biologica) emulsified in CFA at a dose of 200 µg, followed by the administration of pertussis toxin (200 ng; List Biological Laboratories Inc., Colorado, USA) on days 0 and 2 post-immunization. For prophylactic treatment, mice were given febuxostat (0.75 or 1.5 mg/kg body weight per day; Teijin Pharma Ltd., Tokyo, Japan) from day 10 post-immunization to the end of the experiment. For therapeutic purpose and in NOD/ShiJcl mice, febuxostat was administered from day 20 post-immunization. Control animals were given water. Mice were monitored daily for clinical signs of EAE, graded on a scale from 0 to 5 as follows: 0, normal; 1, limp tail; 2, hind limb paresis; 3, hind limb paralysis; 4, paraplegia; 5, moribund [25]. All experimental procedures were approved by the Institutional Animal Care and Use Committee of Osaka University Graduate School of Medicine. All possible efforts were made to minimize animal suffering and limit the number of animals used.

### Histology and Immunohistochemistry

At days 16 and 40 after immunization, mice were perfused with phosphate-buffered saline (PBS) containing 4% paraformaldehyde (PFA). Lumbar spinal cords were dissected and immersed in 4% PFA overnight and then transferred to 30% sucrose overnight and embedded in optimal cutting temperature (OCT) compound and frozen in liquid nitrogen. Frozen sections were cut at a thickness of 10 µm and were processed for immunofluorescence. Sections were incubated with one of the primary antibodies e.g., rabbit anti-iNOS (inducible nitric oxide synthase), mouse anti-Iba1, rabbit anti-XO (Abcam; Cambridge, MA, USA), mouse anti-GFAP (glial fibrillary acidic protein) combined with Alexa Fluor 433 (Cell Signaling Technology, Beverly, MA, USA), rat anti-F4/80 (eBiosciences, San Diego, CA, USA ) overnight at 4°C, followed by incubation with appropriate secondary antibodies. For histological and immunohistochemical analyses, the spinal cord was embedded in paraffin after perfusion with 4% PFA. Axonal loss and demyelination were assessed by staining with Bielschowsky’s silver and Luxol fast blue, respectively. Semi-quantitative histological analysis of inflammatory cellular infiltration was performed according to the following scoring system: 0, no inflammation; 1, cellular infiltrates only in the perivascular areas and meninges; 2, mild cellular infiltration in the parenchyma; 3, moderate cellular infiltration in the parenchyma; 4, severe cellular infiltration in the parenchyma [26].

### Cell Culture and Treatment

The murine microglial cell line, GMI-M6-3 kindly provided by Prof. M. Sawada (Nagoya University), was grown in Minimum Essential Medium (Sigma) containing 10% fetal bovine serum (FBS; Sigma), 1% penicillin-streptomycin (PS; Sigma), 5 µg/ml insulin and 0.2% glucose [27]. The murine J774 macrophage cell line was grown in Dulbecco’s modified eagle’s medium (DMEM) containing 10% FBS, 1% PS [28]. Cells were stimulated with LPS (lipopolysaccharide) or LPS+IFN-γ (interferon gamma) or anti-CD40 antibody+IFN-γ with or without febuxostat at different concentrations for 24 hours. Supernatants were collected for NO (nitric oxide) detection, and the cells were used for iNOS and XO staining. NO was measured using the Griess reaction (Cayman Chemical, Ann Arbor, MI, USA).

### XOR Activity and Oxidative Stress Assay

Frozen tissues were homogenized with potassium phosphate buffer, pH 7.4, containing 1 mM ethylenediaminetetraacetic acid (EDTA) and protease inhibitors. The homogenates were centrifuged at 12,000 rpm for 15 min at 4°C, and supernatants were used for the assay. For XOR activity assay, supernatants were co-incubated with 50 µM pterin solution or pterin with 50 µM methylene blue solution to assay XO or both XO and XDH activity, respectively. After 60 min incubation at 37°C, fluorometric assays were performed to calculate the production of isoxanthopterin in the absence or presence of febuxostat (1 µM) in order to subtract the background. XDH activity was determined by subtracting XO activity from XOR activity. Activity of purified XO derived from buttermilk (Calbiochem, Merck, Darmstadt, Germany) and protein concentration were measured and used to normalize the sample activity [29]. The measurement of thiobarbituric acid-reactive substances (TBARS) was based on the formation of malondialdehyde as assessed by the TBARS assay kit (Cayman Chemical) according to the manufacturer’s instructions.

### Plasma Uric Acid Level

Plasma uric acid levels were determined by an enzymatic method based on the uricase-peroxidase system (autosera S UA, Sekisui Medical Co., Ltd., Japan).

### Real-time PCR

RNA was extracted from lumbar spinal cord with Isogen (Nippon Gene Co., Ltd., Tokyo, Japan), then cDNA was prepared with SuperScript® III First Strand Synthesis III (Invitrogen) and was used as a template for real-time PCR. The expression of iNOS and XO was quantified with specific primers (Sigma-Aldrich): mXO forward (5′ AAAGGACCAGACGATTGCTCC), reverse (5′TCACACGTTCCCCTTCAAAAC); miNOS forward (5′ GCAGAGATTGGAGGCCTTGTG), reverse (5′ GGGTTGCTGAACTTCCAGTC). β-actin forward (5′ GATGACCCAGATCATGTTTGA), reverse (5′ GGAGCATAGCCCTCGTAG) was used as internal housekeeping normalization target.

### Statistical Analysis

Student’s t-test was used for two groups comparisons (semi-quantitative analysis of iNOS and XO staining, relapse rate, TBARS). One way ANOVA followed by the Tukey-kramer multiple comparisons test was used to compare results from Griess reaction, XO activity assay, iNOS relative expression. One way ANOVA and student t-test were used to compare data from clinical and histological scores.

## Results

### Xanthine Oxidase Expression is Increased in the CNS of EAE

**Figure 1:**
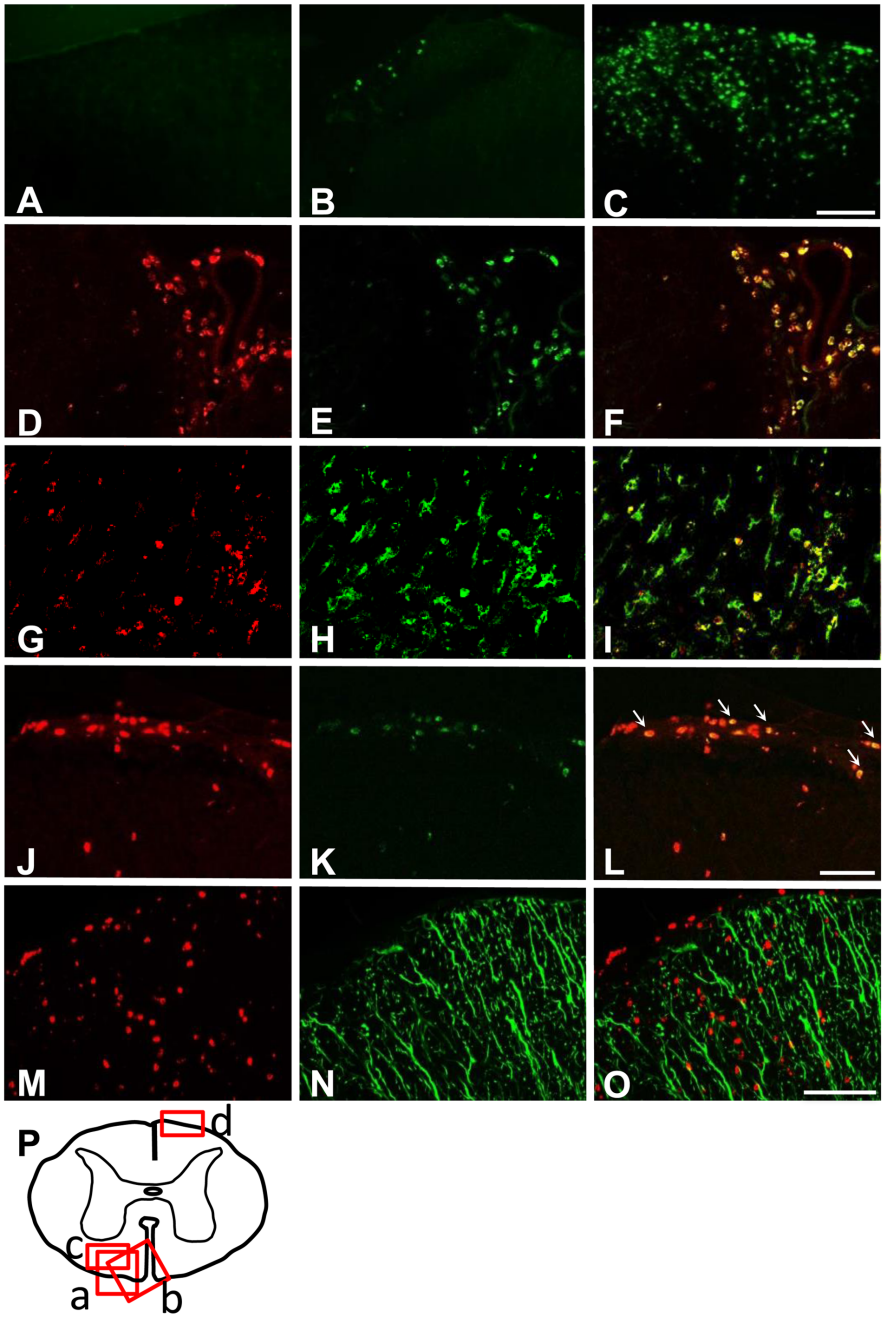
The expression of xanthine oxidoreductase is increased in the CNS of EAE mice. Representative sections of spinal cords from naive mice (A) and non-treated EAE mice (B–O). XOR expression in the spinal cord of naïve mice (A), EAE mice at day 8 post-immunization (B) and at the peak of disease (C) are shown. Scale bar: 100 µm. (D–F) Co-localization of XO-immunoreactivity (D) with F4/80-positive cells (E). Merged image is shown in (F). (G–I) Co-localization of XO-immunoreactivity (G) with Iba1-positive cells (H). Merged image is shown in (I). (J–L) Co-localization of XO-immunoreactivity (J) with CD3-positive cells (K). Merged image is shown in (L). Arrows indicate double-labeled cells. Scale bar: 50 µm. (M–O) Double immunofluorescent staining, showing no co-localization of XO-immunoreactivity (M) and GFAP-positive cells (N). Merged image is shown in (O). Scale bar: 100 µm. P) Schematic diagram of spinal cord with the insets showing the location of the corresponding figures. a) for A–C and M–O; b) for D–F; c) for G–I; d) for J–L.

XOR activity is enhanced under various inflammatory conditions, leading to further tissue damage through ROS generation [18], [22]. To examine whether XOR is involved in the pathogenesis of CNS autoimmunity, we investigated the expression of XOR in EAE mice. Immunohistochemical analysis revealed that the XO expression was first detected in a few cells around day 8 post-immunization in the spinal cords and had markedly increased at the clinical peak of EAE, while naïve mice showed no apparent expression (Fig. 1A–1C). This trend was also confirmed by real-time PCR (Fig. S1). The majorities of XO-positive cells were round-shaped cells and were mainly found at the periphery of the spinal cord and the perivascular areas. Most of these cells were double-labeled with F4/80. Some of the Iba-1-positive process-bearing cells, presumably microglia, were also double-labeled with anti-XO antibody (Fig. 1D–1I). A few of CD3-positive cells were also double-labeled with anti-XO antibody, but none of GFAP-positive astrocytes showed the immunoreactivity to XO (Fig. 1J–1O). These data indicate that the expression of XO is markedly up-regulated in the context of CNS autoimmunity and that XO-positive cells mainly consisted of infiltrating macrophages and microglia. These data also suggest the possible involvement of XOR in the pathogenesis of EAE.

### Prophylactic Administration of Febuxostat Ameliorates EAE

**Figure 2:**
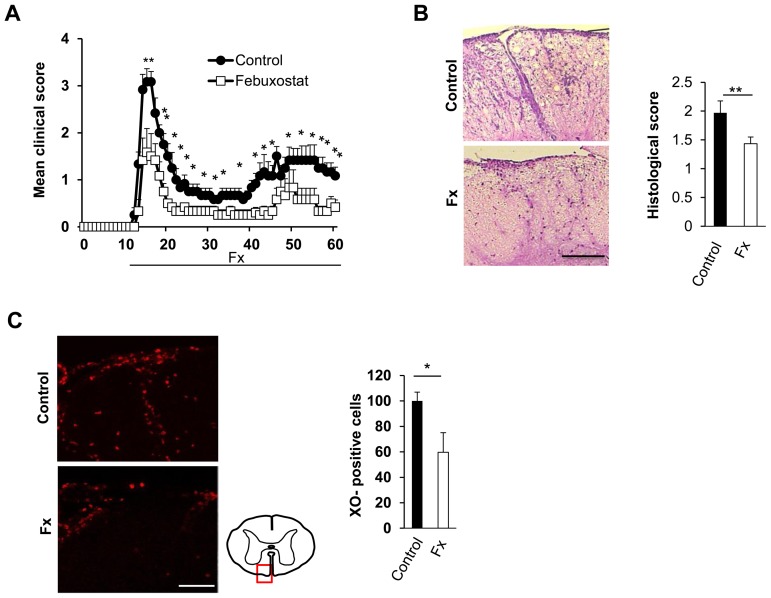
Febuxostat ameliorates EAE. Febuxostat (0.75 mg/kg) or vehicle (water) was administered via water bottle from day 10 post-immunization until the end of the study. (A) Mean clinical scores of control mice (filled circle; n = 7) and febuxostat-treated mice (open square; n = 6) after EAE induction are shown. *p≤0.05, **p≤0.01. Error bars represent the standard errors. (B) Representative H&E staining of lumbar spinal cords on day 16 post-immunization in control (upper panel) and febuxostat-treated (lower panel) groups and the histological score (right panel) are shown. **p≤0.01. Scale bar: 300 µm. (C) XO-immunoreactivities (left panel) of spinal cords of control (upper panel) and febuxostat-treated (lower panel) groups. Schematic diagram of spinal cord shows the location of the corresponding figures. Semi-quantitative analysis of XO-positive cells is shown in the right panel. *p≤0.05. Error bar denotes standard deviation of four mice in each group. Scale bar: 100 µm. Fx: febuxostat.

Because the expression of XOR was increased in EAE, we examined whether the up-regulated XO contributes to the development of EAE. EAE mice were treated orally with a selective XOR inhibitor, febuxostat (0.75 mg/kg body weight), from day 10 post-immunization (before the onset of the disease) to the end of the experiment. The severity of the disease was significantly ameliorated in the febuxostat-treated group when compared with the control group (Fig. 2A). High-dose febuxostat (1.5 mg/kg body weight) did not produce additional efficacy in terms of ameliorating the severity of the disease (Fig. S2). Histological examination of the spinal cords demonstrated that less inflammatory cellular infiltration was observed in febuxostat-treated group when compared with control group. Semi-quantitative evaluation of the cellular infiltration in the spinal cords revealed a significant difference between the two groups (Fig. 2B). Moreover, the increased expression of XOR in the control EAE spinal cord was significantly inhibited by treatment with febuxostat (Fig. 2C). These results suggest that XOR plays a pivotal role in the pathogenesis of EAE.

Peripheral T cell responses were analyzed in both the control and the febuxostat-treated mice to examine whether febuxostat affects the peripheral immunity. No significant differences were observed between the two groups in the production of IFN-γ and interleukin-17 (IL-17) after PLP_139–151_ stimulation of T cells derived from the draining lymph nodes (Fig. S3).

### Febuxostat Inhibits XOR Activity, Oxidative Stress and Glial Activation in the CNS of EAE

**Figure 3:**
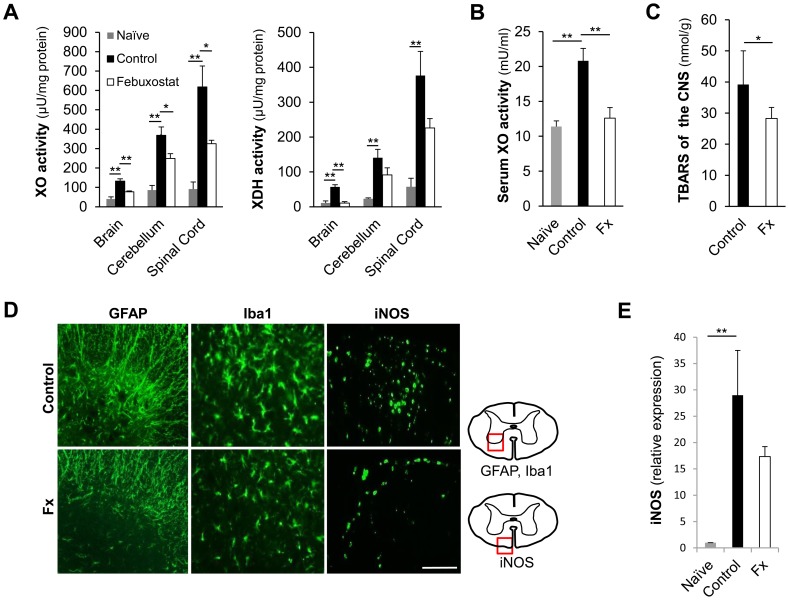
Febuxostat attenuates XOR activities in accordance with the reduction of glial activities. (A) XOR activities in the CNS were measured in naive mice (gray bar; n = 4), control EAE mice (black bar; n = 7) and febuxostat-treated EAE mice (white bar; n = 6). (B) XO activity in the serum obtained from naive mice, control EAE mice and febuxostat-treated EAE mice. (C) Thiobarbituric acid reactive substances (TBARS) were measured in the CNS from control EAE mice (n = 7) and febuxostat-treated mice (n = 6). * p≤0.05, ** p≤0.01. The error bars denote standard deviations. (D) Immunostaining with anti-GFAP, Iba1 and iNOS antibodies of spinal cords at the peak of the disease in control EAE mice (Control) and febuxostat-treated EAE mice (Fx) is shown. Scale bar: 100 µm. Schematic diagrams show the location of the images of the figures. (E) Relative level of iNOS mRNA to that of naïve mice is shown. RNA was extracted from lumbar spinal cords of control and febuxostat-treated EAE mice 15 days post-immunization and from naïve mice. ** p≤0.01. Error bars denote standard deviations (n = 3 in each group).

We next investigated whether febuxostat affects the redox environment in the CNS by inhibiting the XOR activity of the inflamed tissues. A marked increase in the activity of both XO and XDH was revealed in the CNS and in the serum of EAE mice when compared with naive mice. This increase was particularly observed in the cerebellum and the spinal cord, where the inflammatory cells had predominantly infiltrated (Fig. 3A). Febuxostat treatment resulted in significant inhibition of XOR activity in the CNS of EAE mice as well a reduction in XO activity in the serum (Fig. 3A, 3B) when compared with the control group. Serum uric acid level was shown to be elevated after the induction of EAE, and febuxostat-treatment significantly reduced the increase of uric acid (Fig. S4). Moreover, TBARS, which are well-established surrogate markers of oxidation, were decreased in the CNS of the febuxostat-treated group when compared with the control group (Fig. 3C).

In accordance with the amelioration of oxidative stress in the CNS, reduced glial activation suggested by GFAP- and Iba1-immunoreactivities was observed. iNOS-positive cells, predominantly found in Iba1-expressing cells (Fig. S5), were also reduced in the febuxostat-treated group when compared with the control (Fig. 3D). The increase in levels of iNOS mRNA in the control EAE spinal cord was inhibited in the febuxostat-treated group, though this difference did not reach the level of statistical significance (Fig. 3E). These observations suggest that febuxostat reduces oxidative stress in the CNS by inhibiting XOR activity, leading to the amelioration of infiltrating macrophages and activated glial cells.

### Febuxostat Inhibits the Activity of XOR in vitro

**Figure 4:**
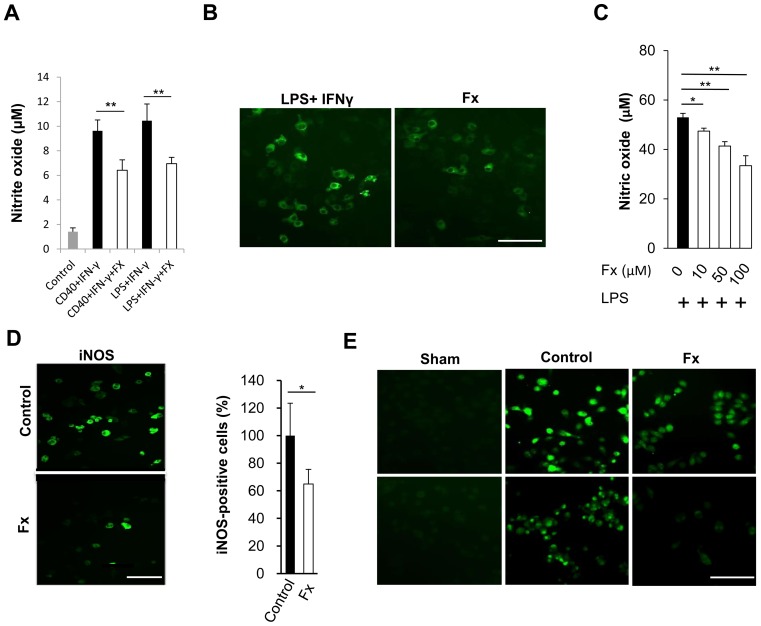
Febuxostat inhibits redox pathways of macrophages and microglia *in vitro*. (A, B) J774 macrophage cells were stimulated with LPS and IFN-γ or anti-CD40+ IFN-γ with or without febuxostat; (A) The supernatant was used to analyze NO expression using Griess reaction. **p≤0.01. (B) The cells were stained with anti-iNOS antibody. (C, D) Murine microglial GMI-M6-3 cells were stimulated with LPS (1 µg/ml) under various concentrations of febuxostat. (C) Concentrations of NO in the supernatant were assessed after a 24-hour incubation period. *p≤0.05, **p≤0.01. (D) The microglia incubated with LPS (control) and with LPS +100 µM of febuxostat (Fx) were stained with anti-iNOS antibody. Representative pictures of iNOS-positive cells are shown in the left panels. The right panel shows the semi-quantitative analysis of iNOS-positive cells in each group. *p≤0.05. The error bars denote standard deviations. (E) J774 macrophages (upper panels) and GMI-M6-3 microglia (lower panels) were left unstimulated (Sham) or were stimulated with IFN-γ+LPS (Control) or IFN-γ+LPS+febuxostat (Fx), then stained with XO antibody. Scale bars: 100 µm.

Previous studies have demonstrated that macrophages and microglia are important sources of ROS and that the oxidative stress induced by these cells is involved in the pathogenesis of EAE and MS [4], [6]. When J774 macrophage cells were incubated with febuxostat under LPS and IFN-γ stimulation, the inhibitory effect of febuxostat on NO production and iNOS induction was observed (Fig. 4A, 4B). Immunocytochemical analysis also showed that the number of iNOS-positive cells was reduced following incubation with febuxostat (Fig. 4B). The inhibitory effect of febuxostat on NO production and iNOS induction was also observed in the microglial GMI-M6-3 cells (Fig. 4C, 4D). The inhibition of NO production by febuxostat was dose-dependent (Fig. 4C). Notably, the suppressive effect of febuxostat on redox responses in J774 cells and GMI-M6-3 cells correlated well with the reduction of XOR expression in these cells (Fig. 4E). These results suggest that febuxostat reduces ROS production by infiltrating macrophages and activated microglia in accordance with a decrease in XOR expression.

### Therapeutic Application of Febuxostat Ameliorates Both Relapsing-remitting and Secondary Progressive EAE

**Figure 5:**
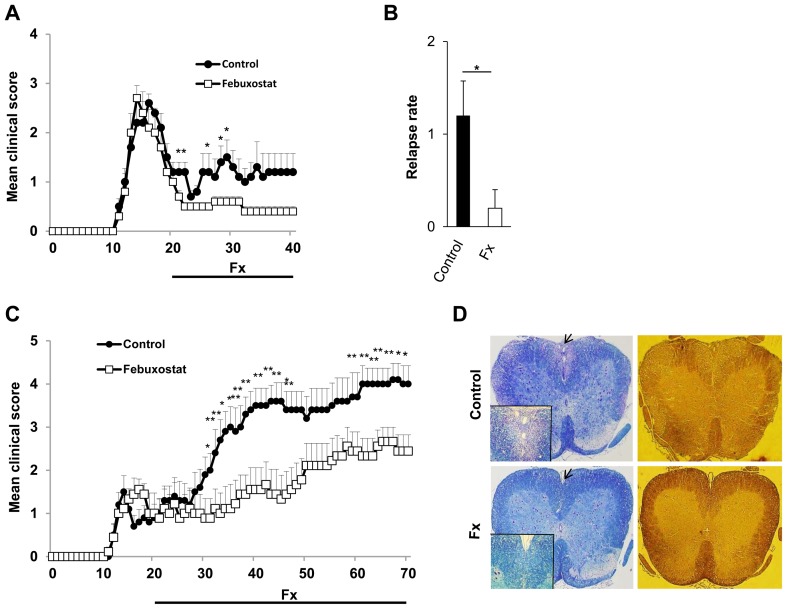
Therapeutic administration of febuxostat ameliorates EAE and prevents axonal and myelin loss in EAE mice. (A) Mean clinical scores of control mice (filled circle; n = 6) and febuxostat-treated mice (open square; n = 6) after EAE induction in SJL/J mice are shown. Febuxostat was given from day 20 post-immunization to the end of the studies.* p≤0.05. (B) The relapse rate was quantified for each group. * p≤0.05. The error bars denote standard deviations. (C) Secondary progressive form of EAE was induced in NOD mice. Mean clinical scores of control mice (filled circle; n = 10) and febuxostat-treated mice (open square; n = 9) are shown. Febuxostat-treatment was started from day 20 post-immunization to the end of the studies. (D) Lumbar spinal cords of control mice (Control) and febuxostat-treated mice (Fx) at day 40 post-immunization were stained with LFB (left panel) and Silver Bielschowsky (right panel) for the analysis of demyelination and axonal loss respectively. Higher magnifications of the areas indicated by arrows are shown in insets.

As we have shown that prophylactic administration of febuxostat ameliorates EAE, we further examined whether it has a therapeutic effect when administered after establishment of symptoms. Administration of febuxostat to EAE mice was started on day 20 post-immunization. The febuxostat-treated group showed significantly less severe symptoms when compared with the control group. In addition, the treated group showed less frequency of relapse (Fig. 5A, 5B). Thus, febuxostat also has a therapeutic effect on the relapsing-remitting form of EAE.

Extensive axonal degeneration and demyelination constitute major determinants of progressive neurological disability in MS, which is coined as a progressive form of the disease. Therefore, we investigated whether febuxostat could also have a therapeutic effect on disease progression in the secondary progressive form of EAE. Induction of EAE in NOD mice with MOG peptide showed a relatively short period of acute CNS inflammation followed by a progressive disease course in the control group, which recapitulates the secondary progressive form of MS [30], [31]. Administration of febuxostat from day 20 post-immunization resulted in a significant reduction in the severity of the disease in febuxostat-treated group compared with the control group (Fig. 5C). Histological analysis showed that febuxostat treatment resulted in apparently less demyelination and axonal loss when compared with the control group (Fig. 5D).

## Discussion

In this study, XOR activity was excessively elevated in the serum and in the CNS of EAE mice, particularly within the cerebellum and the spinal cord, where inflammation predominantly occurs. Moreover, these increases in XOR activity were inhibited by the novel XOR inhibitor, febuxostat, and this inhibition was associated with the amelioration of EAE.

Various studies suggest that activated microglia and infiltrated macrophages are among the main sources of ROS in CNS inflammation in patients with MS [4], [32]. In accordance with this, immunohistochemical studies showed that the strong XO-positive cells in our model were macrophages and microglia. *In vitro* studies suggested that febuxostat effectively inhibits XOR expression by both macrophages and microglia, leading to the reduction of ROS generation. Consistent with these observations, febuxostat inhibited the activation of Iba1-positive cells in our model and furthermore led to the reduction of iNOS expression, which is reported to be enhanced by inflammatory stimuli and further increases ROS generation [33]. These results support a role for XOR within phagocytes in the establishment of CNS inflammation. Though XO immunoreactivity was detected in a few CD3-positive T cells in the CNS, the role in EAE is currently unclear. At least febuxostat seems not to affect the peripheral T cell activation, as it did not inhibit the IFN-γ and IL-17 production in recall assay.

Oxidation of myelin and axons plays a pivotal role in terms of the contribution of ROS to the pathogenesis of EAE [6], [7]. ROS play an important role in myelin phagocytosis, as ROS inhibitors or scavengers reduce the phagocytosis of myelin by macrophages [34]. Oligodendrocytes are highly susceptible to oxidative injury, as they contain high levels of lipid and iron as well as reduced levels of antioxidant enzymes and free radical scavengers [35], [36]. Human oligodendrocyte death associated with ROS induced by XO is reported [37]. ROS also have direct effects on the lipid and protein components of myelin through peroxidation [38]. In addition, a recent study reported that macrophages/microglia-derived ROS and RNS trigger axonal degeneration, and neutralization of ROS and RNS rescues axons [39]. Consistent with these studies, axonal loss and demyelination in our EAE models were markedly ameliorated by febuxostat, correlating with the decrease of ROS and RNS. These results suggest that antioxidant therapy with febuxostat may be effective for both EAE and MS. One possible drawback is that the reduction of uric acid by febuxostat may contribute to the deterioration of the disease, as uric acid is suggested to have a role as antioxidant [40]. However, the amelioration of EAE by the treatment with febuxostat suggests that the reduction of XOR is more important than the reduction of presumed protection by uric acid.

While 85% of MS patients initially present with a relapsing-remitting course, the majority of these patients evolve into a secondary progressive course where disabilities worsen without recovery [41]. The pathology of progressive form of MS is characterized by extensive demyelination, gliosis, and axonal and neuronal losses [42]. Because current disease modifying therapies used for the relapsing-remitting MS (RRMS) are not effective enough for the treatment of progressive form of MS, it is important to identify new therapies for these patients [43]. EAE induced in NOD mice immunized with MOG resembles with secondary progressive MS (SPMS) and is known as secondary progressive EAE [30], [31]. Febuxostat treatment ameliorated the disease progression in this secondary progressive EAE in accordance with the reduction of demyelination and axonal loss. These data suggest that febuxostat may be effective for patients with SPMS.

In summary, the XOR inhibitor, febuxostat, was effective as a therapeutic agent in EAE, likely due to its antioxidant activity. The effect was observed when febuxostat was administered either before or after establishment of symptoms of EAE. In addition, the effect was observed also in secondary progressive EAE. Therefore, XOR represents a novel therapeutic target for the treatment of MS, and febuxostat, which is already approved for treatment of gout in several countries, may be an important agent to modulate XOR activity and improve outcomes in patients with both RRMS and SPMS.

## Supporting Information

Figure S1 **XO mRNA levels in the spinal cords.** Relative levels of mRNA of XO to that of naïve mice spinal cord are shown. Lumbar spinal cords were harvested from EAE mice at days 8 and 15 post-immunization and real-time PCR was performed. p≤0.05. Error bars denote standard deviations (four mice in each group).
(TIF)

Figure S2 **High-dose febuxostat (1.5 mg/kg) does not exert additional therapeutic efficacy in EAE mice when compared with standard-dose febuxostat.** Mean clinical scores of control mice (Control; n = 6) and febuxostat-treated mice (Febuxostat; n = 7) after EAE induction are shown. Febuxostat (1.50 mg/kg) was given from day 10 post-immunization to the end of the studies.* p≤0.05; **p≤0.01. Error bars denote the standard errors.
(TIF)

Figure S3 **Febuxostat-treatment does not inhibit the peripheral immune activation.** Single cell suspensions of lymph node from febuxostat-treated and non-treated EAE mice were prepared 7 days after immunization of mice with PLP _139–151_. Cells were seeded in 96-well plates in 100 µl medium with or without PLP _139–151_. Triplicate cultures were maintained for 72 hours, and IL-17 and IFN-γ in the supernatants were assayed by enzyme-linked immunosorbent assay (ELISA, BD Biosciences).
(TIF)

Figure S4 **Febuxostat reduces the increase of uric acid in the serum of EAE mice.** Uric acid level was measured in the serum of naïve mice and EAE mice (non-treated and febuxostat-treated group) at the peak of the disease. ** p≤0.01; Error bars denote standard deviation.
(TIF)

Figure S5 **iNOS is expressed predominantly by infiltrating macrophages/microglia in the spinal cords of EAE mice.** Double stainings of iNOS with either Iba1 (upper panel) or GFAP (lower panel) are shown. Scale bar = 100 µm.
(TIF)
