# Supplementary figures and images for: Xanthine Oxidase Mediates Axonal and Myelin Loss in a Murine Model of Multiple Sclerosis
Source: PLoS One. 2013 Aug 8;8(8):e71329. doi: 10.1371/journal.pone.0071329 (PMC3738596; doi:10.1371/journal.pone.0071329)

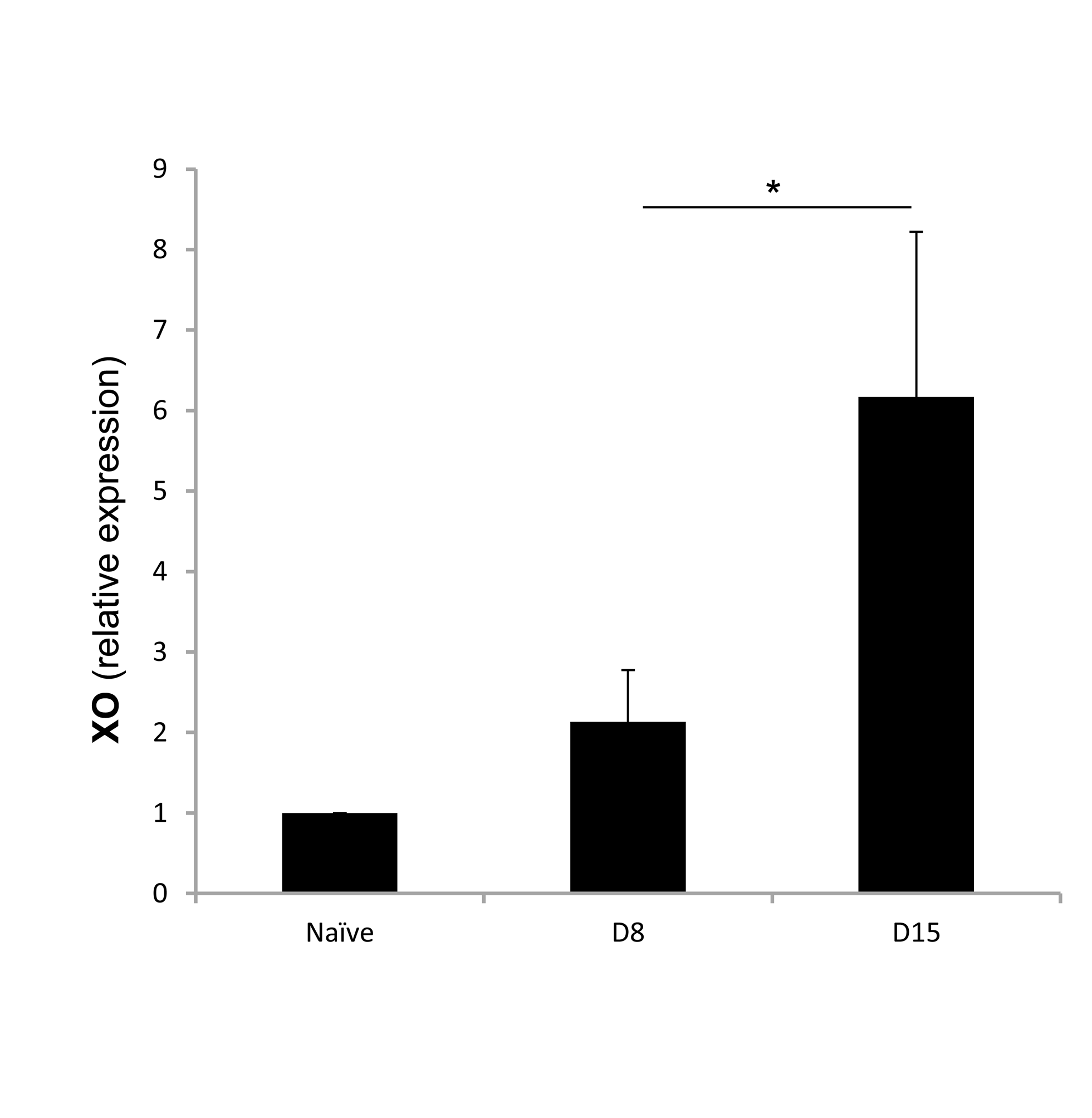

Supplement: Figure S1 — XO mRNA levels in the spinal cords. Relative levels of mRNA of XO to that of naïve mice spinal cord are shown. Lumbar spinal cords were harvested from EAE mice at days 8 and 15 post-immunization and real-time PCR was performed. p≤0.05. Error bars denote standard deviations (four mice in each group). (TIF) [file pone.0071329.s001.tif]

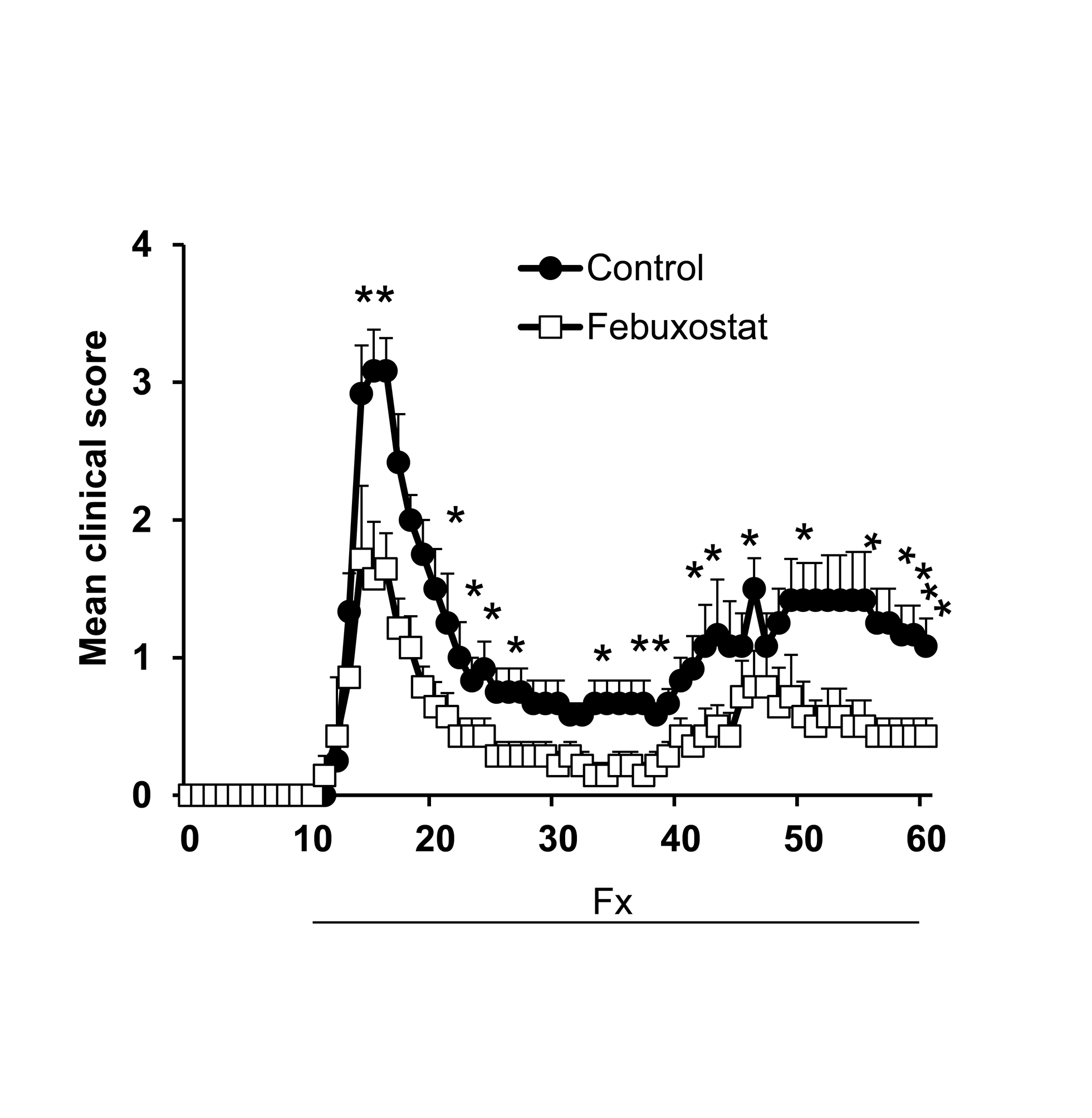

Supplement: Figure S2 — High-dose febuxostat (1.5 mg/kg) does not exert additional therapeutic efficacy in EAE mice when compared with standard-dose febuxostat. Mean clinical scores of control mice (Control; n = 6) and febuxostat-treated mice (Febuxostat; n = 7) after EAE induction are shown. Febuxostat (1.50 mg/kg) was given from day 10 post-immunization to the end of the studies.* p≤0.05; **p≤0.01. Error bars denote the standard errors. (TIF) [file pone.0071329.s002.tif]

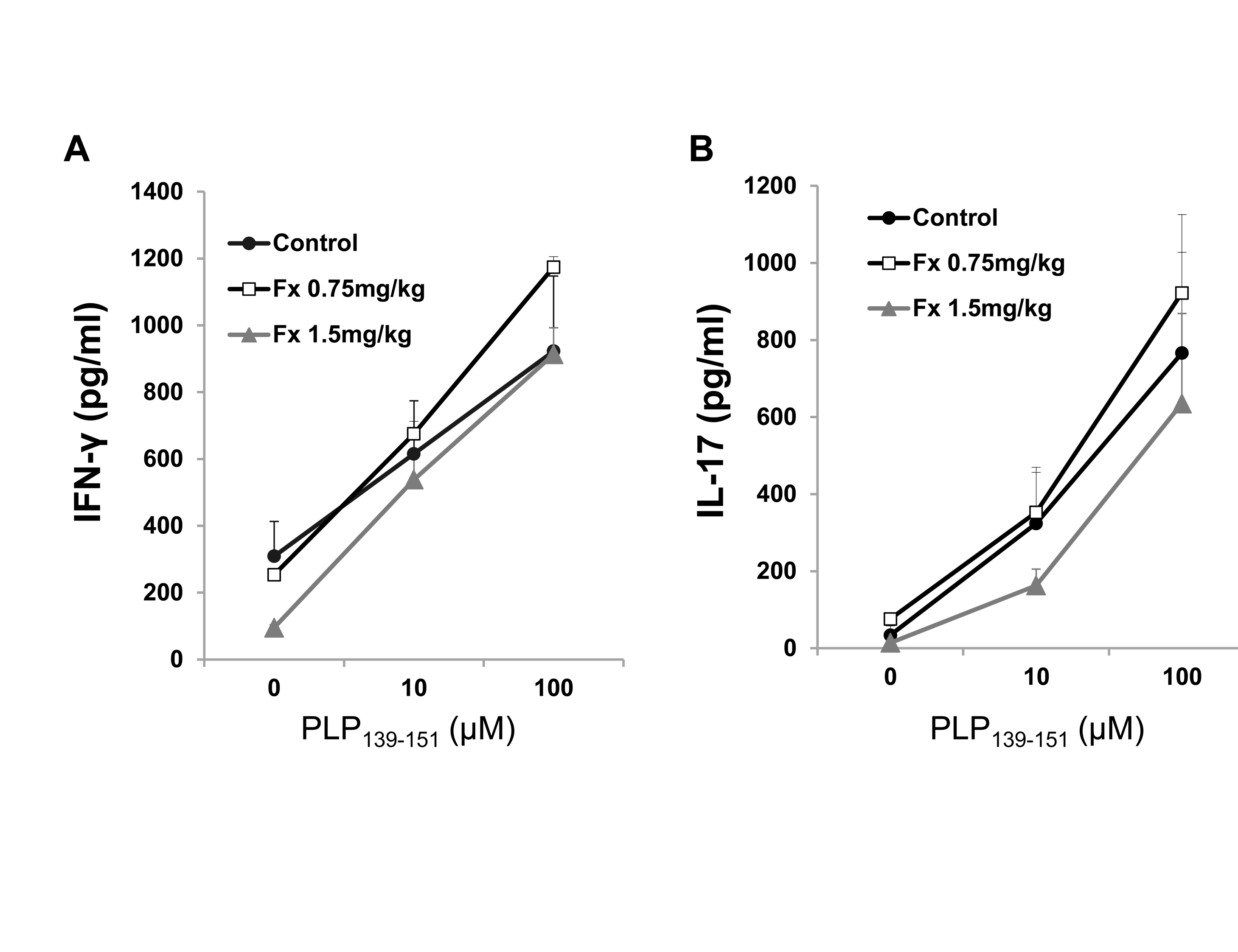

Supplement: Figure S3 — Febuxostat-treatment does not inhibit the peripheral immune activation. Single cell suspensions of lymph node from febuxostat-treated and non-treated EAE mice were prepared 7 days after immunization of mice with PLP 139–151. Cells were seeded in 96-well plates in 100 µl medium with or without PLP 139–151. Triplicate cultures were maintained for 72 hours, and IL-17 and IFN-γ in the supernatants were assayed by enzyme-linked immunosorbent assay (ELISA, BD Biosciences). (TIF) [file pone.0071329.s003.tif]

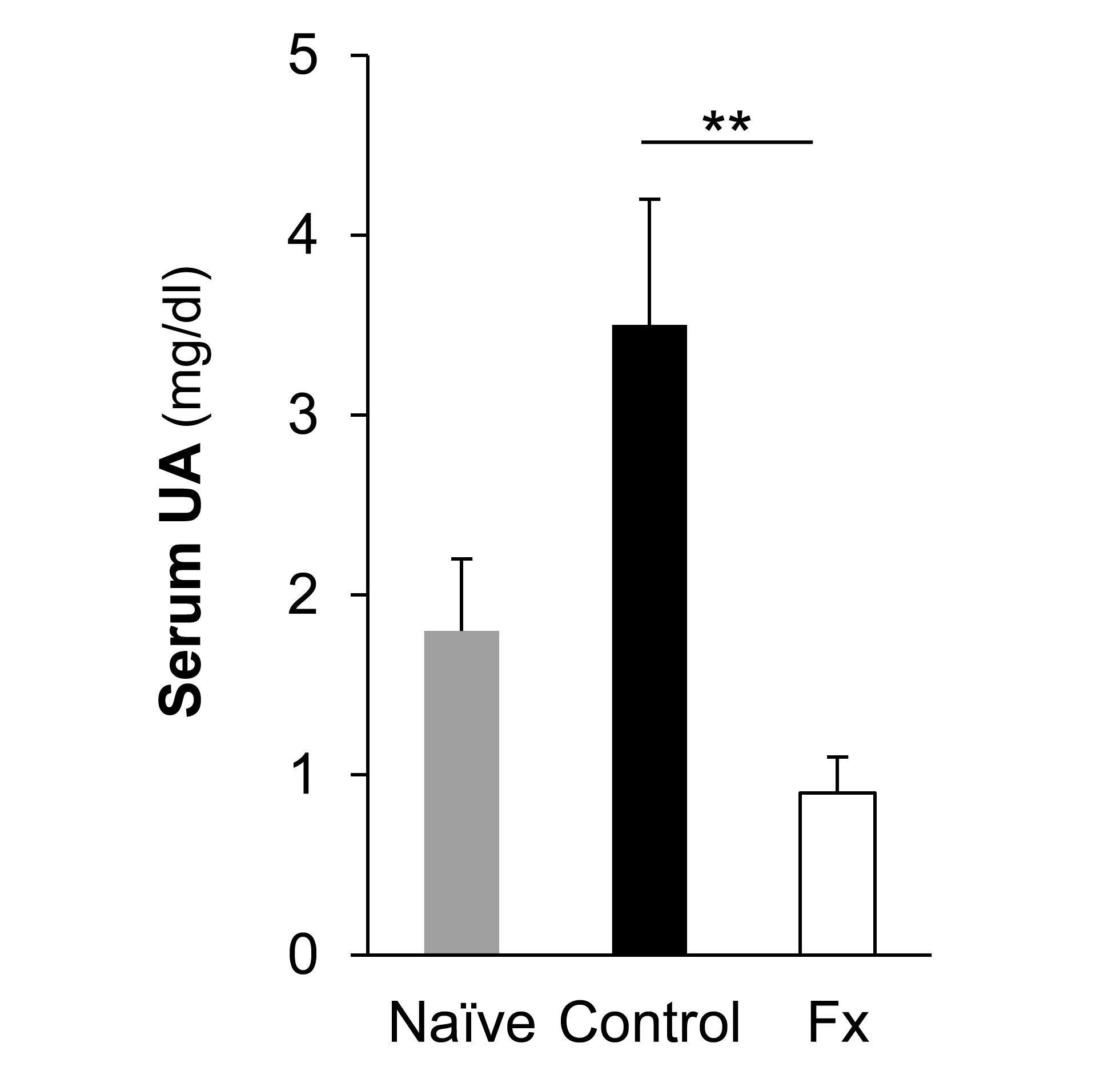

Supplement: Figure S4 — Febuxostat reduces the increase of uric acid in the serum of EAE mice. Uric acid level was measured in the serum of naïve mice and EAE mice (non-treated and febuxostat-treated group) at the peak of the disease. ** p≤0.01; Error bars denote standard deviation. (TIF) [file pone.0071329.s004.tif]

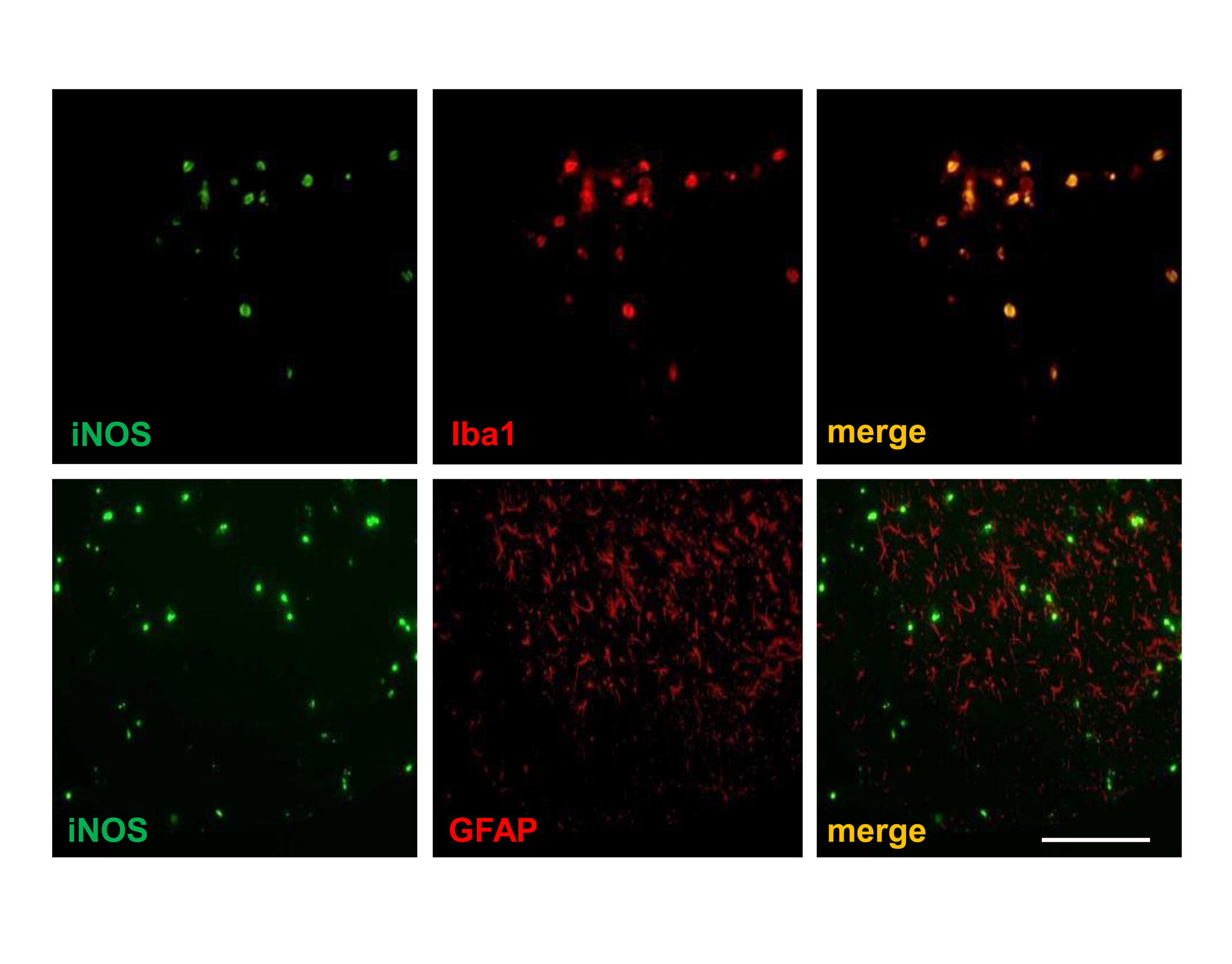

Supplement: Figure S5 — iNOS is expressed predominantly by infiltrating macrophages/microglia in the spinal cords of EAE mice. Double stainings of iNOS with either Iba1 (upper panel) or GFAP (lower panel) are shown. Scale bar = 100 µm. (TIF) [file pone.0071329.s005.tif]
